# QwikMD — Integrative Molecular Dynamics Toolkit for Novices and Experts

**DOI:** 10.1038/srep26536

**Published:** 2016-05-24

**Authors:** João V. Ribeiro, Rafael C. Bernardi, Till Rudack, John E. Stone, James C. Phillips, Peter L. Freddolino, Klaus Schulten

**Affiliations:** 1 Beckman Institute for Advanced Science and Technology, University of Illinois at Urbana-Champaign, USA; 2 Energy Biosciences Institute, University of Illinois at Urbana-Champaign, USA; 3 Department of Biological Chemistry, University of Michigan Medical School, Ann Arbor, USA; 4 Department of Physics, University of Illinois at Urbana-Champaign, USA

## Abstract

The proper functioning of biomolecules in living cells requires them to assume particular structures and to undergo conformational changes. Both biomolecular structure and motion can be studied using a wide variety of techniques, but none offers the level of detail as do molecular dynamics (MD) simulations. Integrating two widely used modeling programs, namely NAMD and VMD, we have created a robust, user-friendly software, QwikMD, which enables novices and experts alike to address biomedically relevant questions, where often only molecular dynamics simulations can provide answers. Performing both simple and advanced MD simulations interactively, QwikMD automates as many steps as necessary for preparing, carrying out, and analyzing simulations while checking for common errors and enabling reproducibility. QwikMD meets also the needs of experts in the field, increasing the efficiency and quality of their work by carrying out tedious or repetitive tasks while enabling easy control of every step. Whether carrying out simulations within the *live view* mode on a small laptop or performing complex and large simulations on supercomputers or Cloud computers, QwikMD uses the same steps and user interface. QwikMD is freely available by download on group and personal computers. It is also available on the cloud at Amazon Web Services.

Nearly 40 years ago, in what was considered the first step of molecular dynamics (MD) simulations applied to biological systems, the dynamics of a folded globular protein (bovine pancreatic trypsin inhibitor) was studied by solving the equations of motion for the atoms with an empirical potential energy function^1^. The results suggested that the protein interior is fluid-like, a result of the diffusional character of local atom motions^2^. Since then, computer simulations of biomolecular systems have grown rapidly, passing from simulating small proteins in vacuum to simulating huge protein complexes in solvent/lipid environments^3^. Building on structural data from diverse experimental sources, today’s MD simulations permit the exploration of biological processes in unparalleled detail^4^. The exponential growth of MD-based investigations is clear when the number of publications indexed at Thomson Reuters’ Web of Science that contain the topic “molecular dynamics” is checked. Figure 1A shows that about 35,000 studies employing MD were published in 2015.

But what exactly is MD and how can it be used? Proteins are not rigid bodies as even the first simulations, and a few experiments before them, were able to show^2^. Dynamics is clearly important for protein function, and discovering the mechanisms underlying function depends on a complete understanding of biological molecules, including their dynamics. Even long before the first protein MD was carried out, Richard Feynman remarked^5^: “*Certainly no subject or field is making more progress on so many fronts at the present moment than biology, and if we were to name the most powerful assumption of all, which leads one on and on in an attempt to understand life, it is that all things are made of atoms, and that everything that living things do can be understood in terms of the jigglings and wigglings of atoms*”. Employing computers and based on a variety of experimental information, MD assists investigation of atomic motion as does no other methodology. The trajectories of molecules are determined mostly by numerically solving Newton’s equations of motion for a system of interacting particles, the molecular atoms. The forces between the atoms and their potential energies are calculated from interatomic potentials in molecular mechanics force fields that are, in effect, huge data bases of molecular properties.

Developed as a simple method in the late 1950’s, MD algorithms evolved greatly, especially for the study of biological systems. All-atom MD simulations, employing classical mechanics, allowed the study of a broad range of biological systems, from small molecules such as anesthetics^6^ or small peptides^7^, to very large protein complexes such as the ribosome^8^, chemoreceptor arrays^9^, or virus capsids^10^,^11^. Hybrid classical/quantum MD simulations allowed the study of enzymatic activity^12^, catalysis^13^, and biological membranes^14^,^15^. All of this was made possible by the development of a multitude of algorithms and MD computer programs closely coupled to ongoing advances in parallel computing and computing hardware. The role of these programs is clearly increasing every year as they become more complex, allowing them to be applied to a plethora of different scientific questions. This importance is reflected by the fact that all these programs are gaining users and, therefore, more citations every year (see Fig. 1B).

Most MD protocols are well established and can be applied to a wide range of investigations, while new protocols continue to be developed, pushing the limitations of MD simulations. Conformational sampling, very long simulations (in the millisecond regime), very large systems (with protein complexes in the megadalton scale), and resolving large structures in real-space are some of these limitations^4^,^16^,^17^. However, even though most protocols are well established, the necessity of understanding the underlying details of these protocols led MD to be viewed for a long time as a demanding technique only accessible to a user with deep knowledge in computational methodologies. Even though the advances in software and hardware in the last decade allowed MD to become accessible to a broader group of scientists, for most of them the necessity of computational methodology knowledge is still an obstacle. Unfortunately, the learning curve to acquire such knowledge is not smooth, requiring years to learn the details of some MD protocols.

A major concern is that, while some novice MD users might feel discouraged by the difficulties, others might end up committing mistakes that are hard to be detected. As with many other techniques, MD is very sensitive to the parameters set by the user, and a mistake in even a simple step might lead to a questionable result. Simple mistakes made while preparing a simulation can easily result in errors in both carrying out and analyzing simulations. However, it is possible to identify a “safe” set of parameters for the vast majority of MD applications. Thus, MD, like any other experimental technique, is turning from a research to a routine laboratory technique, one that could be offered as a laboratory kit, but such a kit has not been made available yet.

Actually, such kit has been already partially presented by a few commercial programs, e.g., MOE and Discovery Studio - BIOVIA. The idea behind these programs, which include modeling, docking, MD and many other computational biology techniques, is to assist users in employing computational biology through automating tedious steps. However, besides being not freely available for research, these programs may lack some of the MD capabilities that some experts need for their work, especially because MD is not the main focus of these programs. Other interfaces, such as CHARMM-GUI^18^, allow a user to prepare simulations, but do not assist in execution and analysis.

Taking advantage of the fact that our group develops two widely employed computational tools for structural biology, namely the simulation program NAMD^19^ and the set-up and analysis program VMD^20^, we developed a new program that connects the two in a robust and user-friendly way to prepare, perform, and analyze MD simulations. The resulting program, named QwikMD, works as a plugin in VMD, taking advantage of the simple menu-oriented point-and-click user interface of VMD enjoyed by hundreds of thousands of registered VMD users. QwikMD offers a smooth learning curve and enables scientists that are not yet MD experts to produce publishable MD simulation results. These simulations are easy to be reproduced, as QwikMD keeps track of every step taken by a user.

Whether carrying out simulations within the *live view* mode on a laptop computer or performing larger and more complex simulations on supercomputers or Cloud computers, QwikMD follows the same steps and provides the same user interface, guiding the user to acquire detailed protein dynamics information. In particular, Cloud computing technologies offer a unique opportunity to make QwikMD, VMD, and NAMD available to the broadest possible user community, including researchers that would otherwise lack convenient access to high performance computing hardware. We have packaged QwikMD, VMD, and NAMD into Amazon Machine Images (AMIs) that give users low-price access through their browser to Amazon Web Services (AWS) to carry out very simple and very advanced simulations in the familiar QwikMD user interface. In doing so, we have effectively converted large-scale MD simulations from a specialized technique applicable only by those with supercomputing access, into a commoditized method that can be purchased and used at whatever scale is needed by any researcher, much like a molecular biology laboratory kit.

The present publication on QwikMD presents the details that make QwikMD a unique tool. Through examples we demonstrate how cutting-edge scientific investigations are carried out in a reliable fashion by means of QwikMD. QwikMD, as is the case for NAMD and VMD, is available free of charge.

## QwikMD Features

**Figure 2:**
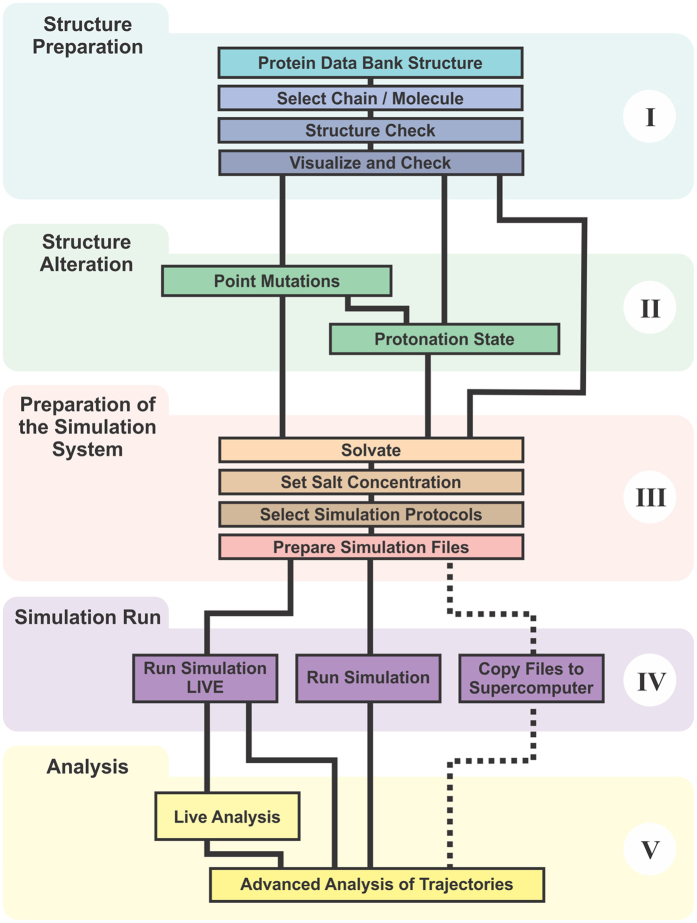
Workflow to prepare, run, analyze, and visualize a molecular dynamics simulation. The workflow is divided into five sections: (I) structure preparation; (II) structure alteration; (III) preparation of the simulation system; (IV) simulation run; (V) analysis. I: An initial three-dimensional structure file containing at least the coordinates of the atoms of the molecule is checked and structural errors are corrected. II: If desired, structural alterations like point mutations or changes in protonation state can be introduced. III: Prior to the MD simulation run, the environment of the molecule and the simulation protocol including simulation time and temperature are defined. IV: The simulation run is executed on a selected computer, e.g., the laptop computer that runs QwikMD, a computer cluster, a Cloud computer or an advanced supercomputer. V: The resulting simulation trajectory can be analyzed and visualized through various VMD tools.

Incorporating the most widely used features of NAMD and VMD, QwikMD provides a graphical user interface (GUI) for the standard MD workflow, represented in Fig. 2. Built on this workflow, QwikMD automates all necessary steps while checking for common errors and ensuring reproducibility of the result by recording each step performed.

The fundamental prerequisite of MD simulations in general, and of QwikMD deployment in particular, is a complete three-dimensional structure at atomic resolution. In QwikMD this structure is first checked for consistency and, if necessary, a user is guided in correcting the structure. Optional alterations like point mutations and changes of protonation state can then be introduced. In order to prepare a simulation, the environment of the structure, such as solvent or membrane, must be specified, structurally determined and the overall simulation protocol and its parameters defined. During the simulation run, NAMD produces trajectory files that contain information about coordinates, energies, and velocities of each atom. These trajectories can be analyzed and visualized with a great variety of VMD tools. The most used analysis tools are fully or partially available through the QwikMD interface, while others can stil be reached through VMD.

Separately in an “Easy/Basic” and “Advanced” tab, for both run and analysis procedures, QwikMD provides a robust and easy-to-use environment for a non-expert user, but also leaves the freedom to adopt variables to specific cases to facilitate and speed up the work of advanced users. Based on the knowledge of the vast NAMD and VMD community of more than 300,000 users, the QwikMD default values are selected to assure robust operation. However, QwikMD is not a black box, it rather provides the user with access to all underlying structures and NAMD configuration files and offers an opportunity to adjust them individually where necessary. While QwikMD guides the user interactively through the process of preparing, running and analyzing an MD simulation it keeps track of every single step, by creating log files, allowing easy reproducibility of the work. In case that the established safe routines fail, which then requires user intervention, QwikMD suggests solutions and strategies for proceeding. These strategies are links to other more advanced VMD and NAMD tools, tutorials, or web-services.

Next, we discuss how the QwikMD concept works in each one of the necessary steps. The present introduction to QwikMD features is mainly directed to an MD novice; for a more advanced reader the methods section of this manuscript provides detailed information on the parameters used by QwikMD.

### Structure check

As stated above, the initial input for QwikMD is a three-dimensional structure of a biological molecule. Several structural biology methods like X-ray crystallography, nuclear magnetic resonance (NMR) spectroscopy, and cryo-electron microscopy are employed to determine a three-dimensional structure at atomic resolution. These structures are usually stored in a freely accessible online database, namely the protein data bank (PDB)^21^, which contains, as of February 2016, over 110,000 structures. Besides coming from experimental methods, the three-dimensional molecular structure of proteins can also be predicted from its amino acid sequence by computational approaches that scan the conformational space applying stochastic algorithms. Common structure prediction programs and web portals are MODELLER^22^, Rosetta^23^, MUFOLD^24^, SWISS MODEL^25^, or I-TASSER^26^. A more detailed description on how three-dimensional structures are obtained and refined, especially for large macromolecular complexes, is available in Goh *et al*.^16^.

**Figure 3:**
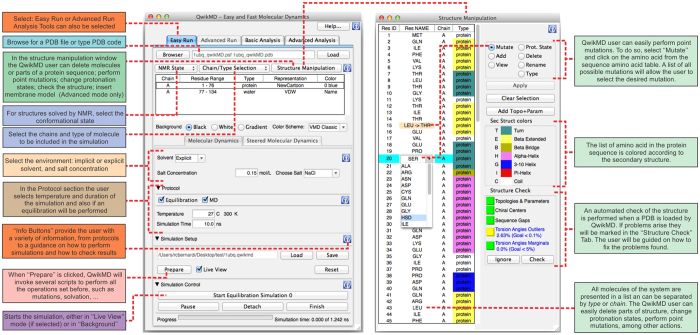
Graphical user interface (GUI) of QwikMD to prepare and run molecular dynamics simulations. Shown is the “Easy Run” tab of the QwikMD GUI and the “Structure Manipulation” window, including all variables that can be adjusted by a user to prepare and run a simulation. In the “Advanced Run” tab the user can select and create further protocols. For convenience, not related to QwikMD’s performance, screenshots were prepared on Mac OS X. QwikMD runs equally well on Linux, Microsoft Windows and Mac OS X.

PDB structures frequently contain more than one copy of the protein one wants to study, due to crystallization effects, and may even include more than one conformational state, when solved by NMR. The QwikMD user can select the molecules or chains of the PDB file that should be investigated by MD simulation, as shown in Fig. 3.

The success of MD simulations depends crucially on the initial structure. In QwikMD, this structure is checked automatically for structural inconsistencies and, if necessary, the user is guided on how to correct such inconsistencies. Quite commonly, parts of a macromolecular structure are simply missing, and must be completed before a simulation is started, except if end pieces are missing, which may just be left out (depending on the biological question of interest). Also, structural components are often not included in PDB files when the respective part of the macromolecule is disordered. This is actually a lucky instance for a modeler as one can readily furnish a reasonable guess for missing structural elements and as the eventual simulation is likely to bear out a high degree of mobility of the respective molecular moiety, offering telling evidence that the disorder is innate and captured by MD. In this case the MD simulation complements experiment.

Finally, a force field information check is performed on the initial structure, testing if all necessary information is available to prepare and run an MD simulation. The force field contains a mathematical formulation of the potential energy describing the multi-atom biomolecular system to be simulated. The force field furnishes, in the form of a huge data base, parameters which describe the detailed mathematical functions accounting for the forces between atoms, calculated actually as respective gradients of a monstrous potential energy expression. Such description allows for the numerous types of chemical properties that together make up the biomolecular systems found inside and outside living cells. Force field parameters are available for standard amino acids, nucleic acids, lipids, sugars, and several other small molecules.

However, for some compounds no parameters are available yet, which is particularly common for drug molecules, especially those under development. In such a case the standard procedure fails and QwikMD points out options that can allow a user to proceed. The easiest way is to delete the non-identified part of the structure, a solution that is usually not desirable as the referred molecule to be deleted might well be biomedically relevant. Thus, the user has to provide QwikMD with the missing force field parameters. If parameters are available, i.e., in the literature, but not yet added to the force field employed, QwikMD offers the option for inclusion of user-provided force field parameters. These parameters can also be obtained from web servers, with varying levels of accuracy. The most accurate method for furnishing missing parameters requires advanced knowledge, namely familiarity with the force field Tool Kit (ffTK) plugin in VMD, to where the user might be ultimately directed. The ffTK plugin assists with the process of parameterization, employing quantum chemistry programs^27^. Force field parameterization, however, is not a simple task and the outcome of the simulation strongly depends on the force field parameters. Therefore, parameterization must be carried out with utmost care, even though it is difficult.

At the end of the structure preparation step, the biomolecules to be simulated should always be visually inspected by the user aided by the wide variety of graphical representation styles, coloring, and atom selection methods provided by VMD.

### Point mutations

Biological processes are often highly sensitive to small structural changes, such as point mutations of proteins. Indeed many human diseases are linked to such point mutations. A common problem in respective scientific studies is that experiments reveal that certain mutations affect cellular processes, but the underlying physical mechanism remains often obscure. MD simulations can play an important role in shedding light on what effects point mutations have on protein behavior. Instead of a typically time consuming and expensive mutational study, MD simulations can be employed to probe structural and dynamical changes induced by mutations before they are actually tried in the laboratory, guiding experimentalists to the most significant mutations. In QwikMD mutations can be easily introduced by selecting the amino acid to be mutated from the amino acid sequence in the “Structure Manipulation” window and selecting the desired mutation (see Fig. 3).

### Protonation states

Biological processes are also highly sensitive to changes in protonation state, even of just a single amino acid. While in experiments the protonation state is indirectly controlled by the pH, in simulations each amino acid has its protonation state set individually. Alterations in protonation states, even though extremely necessary, are usually a more advanced step in MD simulations. In QwikMD, a user can select the protonation state of an amino acid by selecting an amino acid from the sequence and opting for one of its possible protonation states. The procedure is very similar to the one adopted to perform a point mutation.

In the future, QwikMD will include tools that assist in the decision of the protonation state of every amino acid. Such tools, e.g., PROPKA^28^, predicts the optimal protonation state of an amino acid based on a user assigned pH of the medium. Automating protonation state assignment is very challenging and respective tools like PROPKA have a limited precision and will be offered in the future as an option, alerting the QwikMD user to the limitations and advantages of employing such tools. It is however important to mention that, even though limited, the utilization of tools like PROPKA is much better than not taking into account the effect of the local environment on protonation states.

### System and simulation setup

In MD simulations it is necessary to include the environment of the macromolecular system studied. For this purpose, MD simulations are performed in a large set of different conditions, e.g., proteins are simulated in vacuum, in implicit solvent, in explicit water molecules, or in lipid membranes surrounded by explicit solvent at a specific salt concentration. Selecting different environments affects the choice of several input parameters for NAMD runs. These MD input parameters, i.e., how long-range electrostatic interactions are treated, are usually written to a NAMD input file, called the configuration file. In QwikMD, the parameters used as input in this file are selected automatically when a user clicks on the “Prepare” button. The selection of NAMD configuration file parameters is based on all the selections made by the QwikMD user. Table 1 shows some of the NAMD configuration file options assumed by QwikMD according to options specified by a user. Below we describe then in more detail the most common simulation, namely a protein in explicit water solvent. For the advanced reader, more information is provided in Methods.

MD simulations are usually performed in a saline solution of physiological salt strength, with explicit representation of every atom of the solvent. Employing QwikMD, a user can select explicit water molecules and a 0.15 mol/L concentration of NaCl, which is a common physiological condition. Models representing water through its single molecules, so-called explicit models, were developed in the 1980’s to allow the simulation of proteins in a proper thermodynamic bath^29^.

With the environment of water molecules and ions selected, QwikMD automatically takes the necessary steps for a correct simulation in the desired conditions. The NAMD configuration file parameters are adjusted in order to represent properly both environment and conditions of the solvated protein. Employing QwikMD, a NAMD configuration file and all other necessary files are written when the “Prepare” button is clicked and can be inspected as well as altered by a user.

It is recommended to initiate a simulation with a thermal equilibration of the molecular system in the environment conditions. During QwikMD equilibration, an energy minimization is carried out, followed by an MD simulation where the system is heated to a desired temperature (see Fig. 3 for details). During the heating process the positions of the protein backbone atoms are constrained through harmonic potentials in order to maintain the tertiary structure of the protein, while relaxing the side chains of the amino acids and the atoms of the solvent. In the “Advanced Run” tab of QwikMD, a user is also offered the possibility to select which groups will have position restrains, with the most common options already preselected. As in the case of the environment parameters, protocol-dependent parameters like number of steps of each simulation and temperature of a simulation, among others, are written to the NAMD configuration file.

After equilibration a user can initiate the so-called production simulation that produces structure and dynamics, later to be analyzed to deduce the key physical properties of the simulated system. For this purpose a simulation writes the so-called trajectory file, namely, the file of snapshots of the system in time. Each snapshot contains the coordinates and velocities of all or of selected atoms for a given time series, e.g., every femtosecond. The snapshot contains also data of the overall system, like total energy. The level of detail can be specified by the user.

In a usual MD simulation with QwikMD more than one NAMD configuration file will be written. The number of NAMD configuration files will depend mainly on the simulation protocol selected. NAMD configuration files are used by NAMD to perform each simulation step: For example, to perform a system equilibration and a production simulation, two NAMD configuration files will be written, one for the system equilibration and one for the production simulation. A more detailed description of the NAMD configuration file can be found in the NAMD user guide; the detailed description of values used by QwikMD are described in Methods.

Once environment and protocol conditions are selected in QwikMD, and the “Prepare” button is pressed, all necessary files to perform an MD simulation are created, including: the structure of the complete (for example protein and solvent) macromolecular system in the PDB format; the topology information in the PSF format; and the NAMD configuration file. These files are written in a user-defined work directory, where QwikMD creates two folders; a *setup* folder with all steps taken to prepare the system; and a *run* folder with all files necessary to run a simulation.

### Advanced MD protocols

QwikMD is not limited to straightforward MD production runs as described in the example above. Rather, one can perform other MD protocols, e.g., steered molecular dynamics (SMD)^30^ simulations or initiate molecular dynamics flexible fitting (MDFF)^31^,^32^ simulations. In the future most of the protocols available in NAMD will be added to QwikMD. Here we describe how a SMD simulation can be performed employing QwikMD.

In SMD simulations, external forces are employed to explore function and mechanical properties of macromolecules, most frequently proteins. Besides the steps described in the previous sections, in case of SMD simulations the QwikMD user also has to select the chemical groups that has atom position restrained and the chemical groups that are pulled, in a setup similar to a single molecule force spectroscopy using atomic force microscopes or magnetic tweezers. In Applications we describe how SMD is employed to probe mechanical properties of a protein complex.

Steered molecular dynamics is available in both “Easy Run” and “Advanced Run” tabs of QwikMD. In the “Advanced Run” tab, the QwikMD user also has the flexibility to create a new protocol by changing the default values, by combining existing building blocks of the NAMD configuration file, or even by creating their own protocol file from scratch. Such flexibility allows even an expert user of NAMD to benefit from QwikMD.

### Run an MD simulation

**Figure 4:**
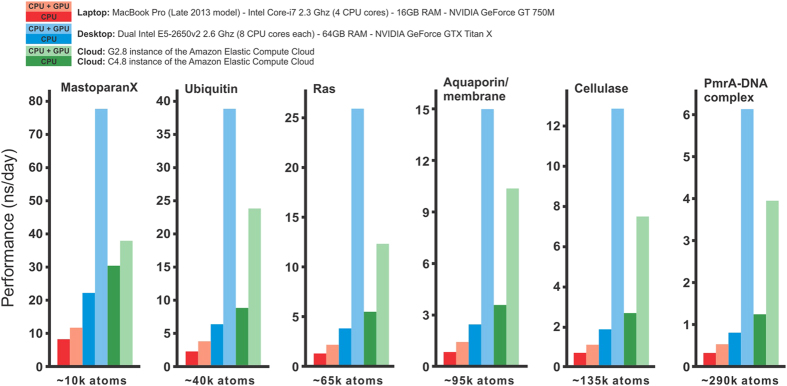
Performance of QwikMD/NAMD simulations on different computer platforms and for different biomolecular systems. Shown are simulations, all set up through QwikMD, of six systems of different sizes (10 K atoms, MastoparanX; 40 K, ubiquitin; 65 K, RAS; 95 K, aquaporin in membrane; 135 K, cellulase; 290 K, PmrA-DNA complex). The simulations were carried out on six different hardware platforms. It is noteworthy that all benchmarks were performed using QwikMD’s “safe” set of parameters. Carrying out the same simulations without QwikMD leads to the same performance as when NAMD is run directly, namely without QwikMD. We note, though, that the use of different sets of parameters optimized for each biological system can result in much better NAMD performance with similar results, in both QwikMD-NAMD runs or direct NAMD runs.

To perform MD simulations QwikMD takes advantage of the widely used program NAMD^19^. In QwikMD, simulations can be either run interactively^33^ with *live view* or in *batch* mode on a desktop or laptop computer. During a simulation in *live view* mode QwikMD provides a progress bar, as shown in Fig. 3, which helps a user to estimate the time till simulation completion. As indicated in Fig. 4, today it is possible to extend simulations, employing a standard workstation with an advanced graphic processing unit (GPU), over timescales that account for a variety of biological processes. In case simulations of very large systems or very long time scales are necessary, the user can perform simulations using Cloud computing (more details in the Cloud computing section below), or on a local computer cluster or on a remote supercomputer. In the case a cluster or supercomputer is employed, the files generated by QwikMD, in particular the *run* folder, need to be transferred to the computer where the simulation is carried out. After the simulations are completed QwikMD can then be employed to perform the trajectory analysis.

The question over which time period an MD simulation should be performed to make sure dynamical properties or processes of interest are sampled sufficiently has frequently no easy answer. Even though MD has always been viewed as a general sampling method^17^, biological molecules are known to have rough energy landscapes^34^ that can make a biological process a very rare event. While some phenomena like side chain flipping and water dynamics occur on a picosecond timescale, others take place only on a nanosecond to microsecond scale, such as secondary structure formation or fast conformational changes. As shown in Fig. 4, these timescales can be easily reached for a variety of highly relevant biological systems and are no longer restricted to a small portion of the scientific community with access to supercomputers, while larger systems might need the usage of enhanced sampling methods^17^, which are available in NAMD but are not described here. Furthermore, many key molecular processes in living cells take milliseconds or longer and, presently are out of reach of straightforward MD simulations on general purpose computers, but can often be described through advanced sampling methods like transition path sampling^35^ (when prior and post states are structurally known or can be modeled) or Markov state modeling when dynamic information can be gathered through many short trajectories^36^.

### Analysis tools

A significant challenge to MD simulations is that there is usually a large amount of data generated, considerably more than typical experimental data. Several tools are available to perform a wide variety of analysis of the extensive MD data. VMD is a powerful tool for analysis as it contains several of the most used algorithms while it also creates interfaces to third party programs employed for trajectory analysis. For any tool that aims to help MD users carrying out actual simulations it is crucial to also enable the means to analyze the resulting simulation data^37^,^38^,^39^,^40^. Beyond simply understanding the static structure derived from experiment or the starting and end points of a simulation, much more information can be gleaned from characterizing the evolution of the atomic coordinates of a biological molecule captured in system trajectories.

**Figure 5:**
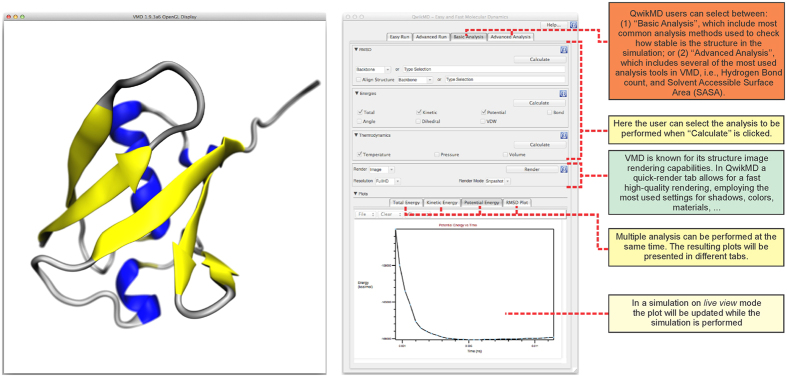
Graphical user interface of QwikMD for analysis and visualization of molecular dynamics simulations. On the left-hand side the molecular visualization (OpenGL) window of VMD is shown, displaying a ubiquitin structure in cartoon representation. In the middle, QwikMD’s “Basic Analysis” tab is shown, that includes tools to plot progression of energies (right side, bottom), or root mean square displacement (RMSD), which are basic criteria to analyze whether a simulation is thermodynamically equilibrated. Furthermore, this tab allows rendering of images of the structures. “Advanced Analysis” tab includes tools employed to plot, among others, the root mean square fluctuation (RMSF), or the behavior of secondary structure over the course of a simulation. Some analysis tools also show their results in the molecular graphics window of VMD, for example, hydrogen bonds when the number of hydrogen bonds is analyzed. All tools in QwikMD’s “Basic Analysis” tab and a few in “Advanced Analysis” tab can also be employed in *live view* mode, showing results while running a simulation. Plots created employing QwikMD can be easily exported. For convenience, not related to QwikMD’s performance, screenshots were prepared on Mac OS X. QwikMD runs equally well on Linux, Microsoft Windows and Mac OS X.

Trajectory analysis refers to analyzing a large set or time series of 3-D positions and their derived properties. QwikMD greatly simplifies the use of such analysis tools provided in VMD by guiding a user to the most broadly used analysis tools, as shown in Fig. 5. Analysis commonly employed as an initial check of the MD simulation, such as a check of root mean square deviation (RMSD) and energetic data are offered in the “Basic Analysis” tab of QwikMD. Analyses that are employed on a case-by-case basis, such as hydrogen bond analysis and solvent-accessible surface area, are present in the “Advanced Analysis” tab of QwikMD. Some of the analyses, i.e., all the ones present in the “Basic Analysis” tab, can also be performed in the interactive *live view* mode of QwikMD, which is especially instructive for novices and for biochemistry teaching purposes.

### Cloud computing

The role of cloud computing in high-performance computing is rapidly increasing today and one can easily predict that it will play a huge role in molecular modeling as the platform is ideally suited for many modeling tasks, both from price and performance point of views. Several Cloud services are available, e.g., from Amazon and Microsoft, offering a wide-range of hardware at an equally wide-range of prices. Employing Cloud computing services, a broad community of researchers can access high-end computing facilities mostly at low cost. On Amazon, Cloud computing services are part of the Amazon Webservices (AWS), which gives remote access to computing hardware configurations, so-called “instance types”.

AWS offers, according to the computational power of the instance type, different sets of “on-demand” prices. The cost of production runs depends on a wide-range of variables, and significantly reduced prices can be obtained by using the so-called “spot pricing”, wherein a user bids on compute cycles and their jobs are run when the price falls below their bid price. For instance, as of February 2016, a whole 100 ns MD simulation of a system with roughly 300,000 atoms costs about US$ 500.00. For experimental groups, which do not require frequent use of MD simulations and do not have access to supercomputer centers, the price of this type of simulation is relatively low compared to the market price of a high-end desktop computer with GPU. However, the strongest argument for Cloud computing for experimental groups is that it does not involve any system administration expertise.

We have packaged QwikMD, VMD, and NAMD into Amazon Machine Images (AMIs), a service of AWS that offers “virtual machines” to users worldwide. QwikMD AMI is a linux-based virtual machine where VMD, NAMD and QwikMD are installed and optimized to give a user the same environment as running these programs on a local computer like their laptop computer. To employ QwikMD using Amazon Cloud, a user needs to access the AWS website (aws.amazon.com) and create an account. With this account one can start any virtual machine available at the AWS portal. When started, QwikMD AMI, as any other AMI virtual machine, is accessed using a remote visualization software that must be installed in a laptop or desktop computer, or even in a portable device.

A more detailed and up-to-date description and overview of the best cost-benefit strategies together with a video tutorial, describing how to use QwikMD together with Cloud computing, is provided on QwikMD’s website (http://www.ks.uiuc.edu/Research/qwikmd/).

## Applications

MD simulations are having a profound impact on molecular cell biology and biomedicine. From physical chemical properties of proteins, to the development of drugs, to the fabrication of novel biomaterials and creation of bio-based renewable energy sources, MD simulations are helping researchers to achieve fundamental understanding of living organisms^4^. To show how QwikMD is employed in preparation of state-of-the-art MD simulations, we selected as examples four systems that are briefly discussed here, ordered by increasing complexity, and presented in Supplementary information as step-by-step tutorial figures.

For the first system, namely **HIV-protease**, MD simulations investigated the drug resistance mechanism of this enzyme and suggested single mutations, which drastically affect protease activity^41^,^42^,^43^. To perform simulations of HIV-protease mutants with QwikMD, very few steps are necessary; indeed a novice to MD simulations can prepare, perform, and analyze the simulations in easy steps. In Supplementary Figure S2 we outline how to prepare and perform simulations of HIV-protease mutants.

The second system involves another family of enzymes, namely **glycoside hydrolases** (GH). These enzymes play a role in producing biofuel from agricultural waste and in the digestion of fibrous foods by bacteria in the lower human gut^44^. MD studies have shown in atomistic detail how GH enzymes work, revealing their inhibition mechanism, a well-known problem in the biofuel industry^45^,^46^. To perform simulations of GHs, the user must specify mutations and changes in protonation state, which is easily done in QwikMD, as outlined in Supplementary Figure S3. Regarding the enzyme’s substrate, QwikMD automatically takes all the necessary steps to correctly account for carbohydrate chains in the catalytic site of GHs, actually a rather complex modeling step.

In two other examples, processes at cellular membranes are studied. Using QwikMD the user prepares in the third example a simulation of a molecule in water near a lipid membrane surface. From biomarkers to drugs, molecules are known to interact with membrane surfaces. As example we outline in Supplementary Figure S4 the steps in which a QwikMD user places a **flavivirus**’ **peptide** near a cell membrane. This peptide is known to cause cell membrane rupture in dengue, yellow fever, and West Nile viruses^7^.

The fourth example deals with the simulation of the trans-membrane water channel **aquaporin**^47^, as outlined in Supplementary Figure S5. Construction of membranes and of membrane-protein complexes with QwikMD is extremely simple. By using a box to represent the membrane, QwikMD allows a user to move this box to the position where the user intends to position membrane, solvent, and protein. When the system is prepared by QwikMD, all the necessary files to perform the simulation of the protein channel - lipid membrane - water solvent system are available for inspection and execution.

### The role of MD visualization

The use of molecular visualization to explore macromolecular processes in biophysics at the atomic level is critical. Visualization offers a route to understanding the physical mechanism underlying cellular processes. Visualization can convey even complex insights about such mechanisms to a broad audience in a comprehensible manner. VMD is one of the most advanced tools for molecular visualization; VMD is used for displaying, animating, and analyzing large biomolecular systems using 3-D graphics^48^. As one example of how visualization can play a significant role in scientific questions, we examine the simulation of a bacterial ultrastable protein-protein interaction. The respective simulation can be readily prepared and analyzed in QwikMD. QwikMD also assists the user in the visualization of simulation results by automatically selecting optimal visualization options, as shown in Fig. 5.

**Figure 6:**
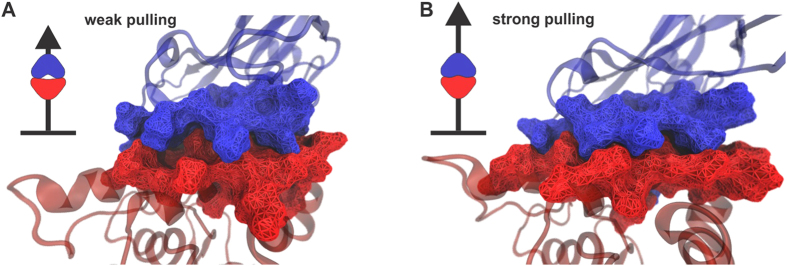
Illustration of the Cohesin-Dockerin contact forming a catch bond. Shown is in this figure the result of a steered molecular dynamics simulation of a complex of Cohesin (blue) and Dockerin (red) set up in QwikMD. (**A**) shows the initial state of the simulation, on the top-left in a schematic form and on the right as visualized with QwikMD (Cohesin and Dockerin are visualized at atomic resolution through their surfaces). One can recognize the two proteins form a loose complex. (**B**) shows the steered molecular dynamics simulation at a later stage, again on the top-left in schematic form and on the right in its visualized form. In the steered molecular dynamics simulation the two proteins are pulled diametrically apart as indicated in the schematic figures. Surprisingly, the loose complex in (**A**), existing as long as the complex is pulled apart with weak forces, is not readily separated through the application of a stronger pulling force, but rather the complex becomes tight upon strong pulling. This behavior is referred to as a catch bond mechanism.

Combining state-of-the-art single-molecule atomic force microscope techniques and steered molecular dynamics (SMD) simulations a protein complex was shown to withstand forces of 600–750 pN^49^, making it one of the strongest bimolecular interactions reported. Amazingly, the protein complex involving a protein called Cohesin and a protein called Dockerin, develops its strong cohesion only when the proteins are pulled apart^49^,^50^. The mechanism underlying this behavior is presented in Fig. 6.

Simulations and experiments combined suggested that a catch bond mechanism is responsible for the complex’s remarkable stability under force^49^. It is remarkable that the necessary advanced simulations can be set up, executed, and visualized in QwikMD. In Supplementary Figure S6, we show step-by-step how the respective simulations are set up in just a few minutes employing QwikMD. The advanced image rendering tools of VMD allow creation of high quality images that help to explain the results^48^,^51^, as shown in Fig. 6.

## Perspective

Through the combination of state-of-the-art computing hardware and advanced software molecular dynamics simulations emerged today in the role of a computational microscope^16^, and QwikMD makes this microscope available to a much broader community of users. MD simulation has established itself as a reliable tool for the study of the structure and dynamics of large protein complexes within realistic cellular environments^4^. As MD plays an ever-increasing role in structure biology, tools like QwikMD will become increasingly necessary. It is not realistic to assume that every potential user of MD simulations can be a computer and molecular physics expert. We envision that QwikMD will support users greatly through more complex MD protocols, which currently require very time consuming preparation, such as: free energy perturbation; adaptive biasing force; umbrella sampling; replica-exchange molecular dynamics; constant-pH simulations.

Advances in computer hardware and in software tools like QwikMD, NAMD, and VMD will make MD simulations more valuable and accessible to experimental laboratories. QwikMD enabled molecular dynamics simulations will depend strongly on the evolution of computing hardware. While large supercomputers are reaching the “exascale era”, allowing some groups to simulate whole small cells in atomistic detail, the development of new GPU accelerators, many-core CPUs, and remote visualization platforms will allow the whole cell biology community to take advantage of MD tools for proteins and large protein complexes. Here, we discuss some of the expected hardware evolution for the next few years and the implications to molecular dynamics.

### Hardware evolution

Over the next few years, it is widely expected that continued advances in the development of commodity microprocessors will yield many-core CPUs and new generations of GPUs raising peak performance by as much as a factor of ten. While these advances will provide a tremendous boost in the scientific computing capabilities of individual microprocessors, it is unclear whether concomitant technological advances will be made integrating these processors into efficient parallel computers.

One conclusion drawn from observation of ongoing hardware trends is that the scope and performance of simulations that run on individual or tightly-coupled CPUs and GPUs can be expected to continue improving at a rapid pace, and that one can expect this to be of greatest benefit for researchers studying moderate-size macromolecules on modest computing systems such as desktop workstation and laptop computers. We feel that QwikMD, in combination with NAMD and VMD, will help a broad range of users exploit this computing hardware territory to perform MD simulations that would have required a supercomputer just a year ago.

While we envision a majority of users would apply QwikMD to molecular systems of 500,000 atoms or less, an ongoing increase in the availability of atomic-detail experimental structures for large biomolecular systems such as viruses, genetic expression systems or the nuclear pore complex, will drive research toward larger simulations. To make QwikMD more directly applicable to such systems without computing, storage, analysis, and visualization limitations imposed by personal computers or laptops, we have begun to adapt QwikMD, VMD, and NAMD for execution in Cloud computing environments such as Amazon EC2. Using the Cloud, a complete work environment containing all required software, and arbitrarily large computing and storage resources can be made available on-demand at low cost to any researcher at a moment’s notice. The use of state-of-the-art remote visualization technologies maintains all of the interactivity and molecular graphics and rendering features normally available on a high-end workstation, but at any geographic location. In the context of MD, one of the key benefits of Cloud computing and similar virtual workstation approaches is the elimination of all of the software installation and system administration work associated with managing clusters of high performance computers, GPU accelerators, and other advanced computing technologies that are not necessarily widely used by experimental labs and other novice MD users that QwikMD is aimed at helping.

Compared to Cloud computing five years ago, price, performance, and convenience of Cloud computing improved substantially, especially with the advances in remote visualization^48^,^52^. As broad use of Cloud computing increases, we expect that the cost will continue to decrease further, and ease of use will continue to increase.

## Conclusion

QwikMD was developed to assist MD novices, especially experimentalists, to overcome the initial learning curve barrier hindering general use of MD simulations. QwikMD connects the widely employed and user-friendly molecular graphics program VMD to the powerful parallel MD program NAMD. A QwikMD user is able to prepare an MD simulation in just a few minutes, allowing rapid studies of point mutations, partial deletions, and even atomic force microscopy experiments. QwikMD facilitates for novices the performance of MD simulations, while it also serves as a guided learning tool. Many “Info Buttons” provide the theoretical background of the underlying procedures carried out in modern MD simulations. At the same time, the log files tracking all steps of the QwikMD user enables simulation reproducibility and the sharing of MD simulation protocols with collaborators. Furthermore, log files provide an easy way to check and discuss the MD protocols inside a group, which can be especially helpful for novices.

With the recent advances in Cloud computing platforms, we choose to make QwikMD Cloud-ready. QwikMD, NAMD and VMD are now packaged into Amazon Machine Images, allowing research scientists access to high-end computer hardware with all necessary software already installed and ready to use. While the costs of such platforms are prohibitive for groups with large demand for computational simulations, where having their own local computer cluster is more cost-effective, Cloud platforms are extremely price effective for groups that do not employ MD on an extensive basis.

In summary, QwikMD is a robust, user-friendly, and freely available software that enables novices to answer a broad range of questions about molecular systems, with applications that range from cell biology to health sciences. Performing both simple and advanced MD simulations interactively, QwikMD automates as many steps as necessary while checking for common errors and enabling reproducibility, allowing the QwikMD user to study basically any biological system for which structures are available. Even though QwikMD was designed mainly as a tool for novices, it meets also the needs of experts in the field, increasing the efficiency and quality of their work.

## Methods

QwikMD, itself a VMD plugin, employs and calls a wide range of other VMD plugins and NAMD scripts developed and established over the past two decades. QwikMD does most of its work preparing simulations using two tools, namely psfgen and autopsf. These tools are responsible for generating key NAMD input files, applying changes in the protonation states, and also for making point mutations, which is achieved by altering the coordinates for the substituted atoms. In the following text, we discuss how QwikMD works behind its simple menu-oriented point-and-click user interface. The concepts presented here are directed to a more advanced reader, however a novice might also find it helpful, as this discussion presents the basic principles behind VMD plugins that are employed for structure checking, preparation, simulation, and workflow logging in QwikMD.

### Structure check

Biomolecular structures, whether obtained from the PDB or from other sources, may contain a variety of structural errors, such as missing residues or incorrect sterochemistry. These errors are mainly connected to the difficulties arising for atomic resolution structures of highly flexible protein regions. QwikMD provides automated tests via its Structure Check plugin to call the user’s attention to any questionable portions of the structure and assist in correcting the identified issues. The user may then apply their biological knowledge, through the Structure Check interface, to obtain proper coordinates that define a better starting conformation of the local structure. In the future, QwikMD will also incorporate and/or interoperate with tools to identify missing residue segments and to assist in predicting the structure of the macromolecule around such segments.

Before an MD simulation can start, force field parameters must be assigned to all atoms in the molecular system. When a PDB file is loaded into QwikMD, the search for force field paramenters is the first step taken. If non-standard residues or non-standard molecular structure are identified, QwikMD highlights these parts and advises a user to take actions to resolve the issue, e.g. by renaming molecules that might present a different name in the PDB and in the CHARMM force field.

### Psfgen and autopsf

QwikMD uses the VMD plugins, psfgen and autopsf, to perform the crucial work of preparing molecular structure files containing all information necessary for a NAMD run. Of these plugins, psfgen serves as the core performing the actual structure generation, whereas autopsf provides a more convenient and flexible interface that frees the user from many tedious, error-prone steps that require expert knowledge if psfgen is run directly.

#### Generation of molecular topology

The psfgen module incorporates the structure-building capabilities of the CHARMM program^53^ into VMD and NAMD by interpreting and applying the molecular topology descriptions contained in CHARMM-format. rtf and .str files. The resulting structural information, contained in a PSF file, is a required input for NAMD simulations. Psfgen is distributed as a compiled module that is loaded at runtime within the VMD and NAMD Tcl interpreters, and it is also distributed with NAMD as a stand-alone program. Psfgen is driven by commands in the Tcl scripting language as described in NAMD’s User Guide.

#### Psfgen requires splitting of molecules into segments

Structure generation in psfgen is based on named segments, each containing numbered residues. The sequence of residue IDs may contain both gaps and alphabetical insertion codes to distinguish residues with the same number, as is standard practice in PDB files. The sequence of residues within each segment may be specified directly, but is typically extracted from a PDB file containing only atoms from that segment. Aliases may be defined to map residue types in the PDB file to those of the topology files. For each segment the user may specify alternate patches for the initial and terminal residues, and change the type of individual residues (e.g., to protonated variants).

Generated segments are accumulated in memory, comprising both per-atom data (name, type, mass, charge) and bonded terms (bonds, angles, dihedrals, impropers, cross-terms). Angles and dihedrals are typically generated based on bonds, while other terms are specified explicitly in the topology file. After segments are generated, additional patches may be applied to further modify individual residues (e.g., to convert RNA into DNA) or to link non-consecutive residues (e.g., disulphide bonds). Some patches require regeneration of angles and dihedrals. The completed structure may be written to a .psf file.

#### Determination of initial coordinates

Once a molecular structure is built, initial coordinates must be determined for all atoms. Coordinates are read by the coordpdb command from PDB files, generally the same files from which the sequence of the corresponding segment is extracted. The segment name, residue ID (not type), and name of each atom in the PDB file are used to find the corresponding atom in the generated structure and the coordinates of the appropriate atom are set to the coordinates in the PDB file. Aliases may be defined to map residue type and atom name pairs in the PDB file to those of the topology files if nonstandard nomenclature has been used. The coordinates of most heavy atoms will be extracted from PDB files, but normally some heavy atoms are missing from the crystal structure, along with most hydrogens.

Psfgen uses the internal coordinate (IC) records in the topology file to guess atom coordinates based on default values for bond lengths, angles, and torsions, repeatedly iterating over atoms with unknown coordinates until there are none left or no new coordinates can be determinated. IC records are not applied if they would generate bond angles less than 45° given the existing coordinates. Hydrogen atoms for which IC records do not exist, are assigned coordinates based on simple heuristics of a tetrahedral or trigonal bonding pattern. Warnings are printed for atoms with “poorly” (i.e., heuristically) guessed or completely unknown coordinates. The coordinates may be written to a .pdb file, with the occupancy field set to 1 for atoms with known coordinates (i.e., coordinates read from a file), 0 for atoms with guessed coordinates, and −1 for atoms without coordinates. When minimizing and equilibrating the simulation the occupancy field may be used to fix or restrain atoms with known coordinates.

#### Handling of multiple structures

Psfgen allows the reading of existing structure files, which are merged into the current structure being built. If merging is not desired, a new structure may be started with the resetpsf command, or both structure and topology files discarded with “psfcontext reset”. Structure and coordinates can be loaded by separate readpsf and coordpdb commands, but if the atoms in the .psf and .pdb files correspond exactly it is safer and faster to bypass the name matching used by coordpdb by reading both files with a single command as in “readpsf struct.psf pdb struct.pdb”.

#### Autopsf automates most aspects of PSF generation

As is often the case with powerful, general-purpose tools, psfgen requires user experience and planning to develop appropriate structure building scripts for a given project. Autopsf provides a simplified interface automating most aspects of each key step in the psfgen workflow, including the identification of segments, atom/residue name aliasing, and application of common patches described above. Autopsf uses heuristics derived from extensive work on biomolecules from the PDB to automate the most tedious and error-prone parts of the psfgen workflow (most notably, assigning and generating separate files for different segments, and doing the necessary bookkeeping to ensure that all chains are properly loaded and patched in the psfgen script). Following a philosophy similar to QwikMD as a whole, autopsf includes safe default settings which are applicable to the vast majority of biological systems.

#### Integration of autopsf with user workflows

The final output from autopsf is a complete, and optionally solvated system, that is fully prepared for simulation using NAMD. For the beginner, the abstraction provided by autopsf, and even reinforced by QwikMD, allows one to generate a reasonable structure without needing to directly write a psfgen script, substantially facilitating system preparation. For the expert user, autopsf may still be of use because it allows automation of common and tedious tasks and prevents simple but potentially serious errors (e.g., accidental omission of a segment or patch). Whatever their level of experience, however, users would do well to remember that the heuristics used by autopsf provide reasonable results in *most*, but not *all*, cases and it is still crucial to inspect the resulting structure to ensure that chain definitions and patching decisions are appropriate. Poor performance or apparently incorrect decisions by the software are more probable for systems that deviate from the designed use case (which is a primarily biomolecular system obtained from the Protein Data Bank^21^).

### Molecular Dynamics protocols

To perform a common MD protocol in explicit water, QwikMD solvates the system in a cubic box and sets its net charge to zero by adding ions. If specified by the user, a salt concentration is added by randomly replacing water molecules. The CHARMM36 force field^54^,^55^ along with the TIP3 water model^56^ are employed to describe the system. During the simulations a 12.0 Å cut-off is applied to short-range, non-bonded interactions, whereas long-range electrostatic interactions are treated using the particle-mesh Ewald (PME)^57^ method. The equations of motion are integrated using the r-RESPA multiple time step scheme to update the short-range interactions every step and long-range interactions every two steps. The time step of integration is set to be 2 fs for all simulations performed. The first nanosecond of the simulations serves to equilibrate systems before the production runs. During this process a temperature ramp is set so that the system reaches the equilibrium temperature slowly. For pulling experiments, SMD simulations^30^ of constant velocity stretching (SMD-CV protocol) are performed, with a pulling speed set by the user. The user must identify both an anchoring residue and a pulling amino acid residue, or group of residues. The procedure is equivalent to attaching one end of a harmonic spring to one end of a protein/domain and pulling on the other end of the spring. The force applied to the harmonic pulling spring is then monitored during the time of the steered molecular dynamics^30^,^49^,^58^.

### Reproducibility and documentation

Due to the countless options available in the configuration file of MD software reproducibility has been a key issue in MD simulations. Workflow management, already available as a tool external to MD software^59^, offers a solution by keeping a log of all steps taken. QwikMD, while guiding the user interactively through the process of preparing, running and analyzing MD simulations, keeps track of every single step by writing two files, a *.qwikMD* file and a *.infoMD* file. Prepared like a *tcl* script, the *.qwikMD* file can be loaded through the QwikMD GUI and contains all information necessary to reproduce the content of both the *setup* and the *run* folders. The *.infoMD* is the more easily readable version of the log file and intended for easy communication of simulation details, containing all the information about how the simulation was prepared, performed and analyzed. These two log files allow the user to exactly reproduce the procedures followed and can subsequently be used guiding work on other biomolecular systems, or in manuscript writing.

The concept of protocol documentation in QwikMD enables reproducibility at the same time as it facilitates sharing MD simulation protocols with collaborators. Groups can use the QwikMD log workflows to control, discuss, and improve scientific progress. For teaching purposes, the log files can be used as a quality control for the work performed by a student. In general, the unified way of documenting simulations makes MD more accessible to a novice, lowering the learning barrier of MD simulations.

## Additional Information

**How to cite this article**: Ribeiro, J. V. *et al*. QwikMD — Integrative Molecular Dynamics Toolkit for Novices and Experts. *Sci. Rep.* **6**, 26536; doi: 10.1038/srep26536 (2016).

## Supplementary Material

Supplementary Information

## Figures and Tables

**Figure 1:**
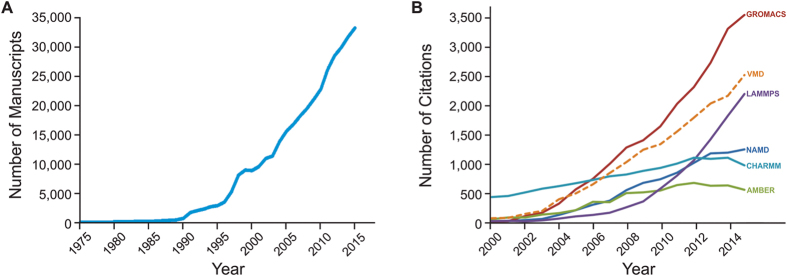
Development of molecular dynamics over the past decades. (**A**) Number of MD papers per year since 1975. The value was calculated using the search term “molecular dynamics” in Thomson Reuters Web of Science, where we filtered the number of publications per year that present MD as a research topic. The curve shows a substantial evolution of MD simulation over the years, even though our search term is likely excluding many publications that present MD results. (**B**) Evolution of the usage of some of the most employed MD tools today. Visual Molecular Dynamics (VMD) is also included as it is one of the most employed set-up and analysis tools. The plot shows the number of citations (Google Scholar) of these programs’ main publications per year. We considered 2 publications for NAMD; 5 for GROMACS; 3 for AMBER; 1 for LAMPS; 2 for CHARMM; and 1 for VMD. The list of publications employed is presented in Supplementary Note S1. The plots show a clear evolution of the whole MD field as well as of the usage of the main MD programs. It is worth noting that the number of citations is underestimated, as many publications employing MD simulation do not cite the MD program in the main text, cite other manuscripts, or do not cite any software. However, this problem affects all programs in a similar manner. Also, many other factors might be influencing the number of citations of the MD programs, e.g., AMBER and CHARMM, having a force field with a similar name, might have “wrong” citations that were intended for the force field. We believe that the number of these and other “wrong” citations is not significantly influencing the plot. Data obtained in February 8, 2016.

**Table 1:** Configuration file settings depending on different simulation environment.

Environment	PBC	Long Range Electrostatics	Constant Area	Remarks
Vacuum	OFF	Cut-off	NA	Dielectric constant 80
Implicit Solvent	OFF	GBIS^60^	NA	Several parameters necessary for Generalized Born Implicit Solvent (GBIS) method
Explicit Water Solution	ON	PME^57^	NA	System is solvated in a cubic box
Explicit Membrane	ON	PME^57^	Available	Available in the “Advanced Run” tab

(PBC: Periodic Boundary Condition; GBIS: Generalized Born Implicit Solvent; PME: Particle-Mesh Ewald).

## References

[b1] McCammon J. A., Gelin B. R. & Karplus M. Dynamics of folded proteins. Nature 267, 585–590 (1977). 301613 10.1038/267585a0

[b2] Karp G. Cell and Molecular Biology (John Wiley & Sons, Inc., 2002).

[b3] Orozco M. A theoretical view of protein dynamics. Chem. Soc. Rev. 43, 5051–5066 (2014). 24709805 10.1039/c3cs60474h

[b4] Perilla J. R. . Molecular dynamics simulations of large macromolecular complexes. Curr. Opin. Struct. Biol. 31, 64–74 (2015). 25845770 10.1016/j.sbi.2015.03.007 PMC4476923

[b5] Feynman R. P., Leighton R. B. & Sands M. L. The Feynman Lectures on Physics: Mainly Mechanism, Radiation and Heat (Addison Wesley, 1963).

[b6] Bernardi R. . Molecular dynamics study of biomembrane/local anesthetics interactions. Mol. Phys. 107, 1437–1443 (2009).

[b7] Mendes Y. S. . The Structural Dynamics of the Flavivirus Fusion Peptide-Membrane Interaction. PLoS ONE 7, e47596 (2012). 23094066 10.1371/journal.pone.0047596 PMC3477123

[b8] Bock L. V. . Energy barriers and driving forces in tRNA translocation through the ribosome. Nat. Struct. Mol. Biol. 20, 1390–1396 (2013). 24186064 10.1038/nsmb.2690

[b9] Cassidy C. K. . CryoEM and computer simulations reveal a novel kinase conformational switch in bacterial chemotaxis signaling. eLife 4, e08419 (2015). 26583751 10.7554/eLife.08419 PMC6746300

[b10] Freddolino P. L., Arkhipov A. S., Larson S. B., McPherson A. & Schulten K. Molecular dynamics simulations of the complete satellite tobacco mosaic virus. Structure 14, 437–449 (2006). 16531228 10.1016/j.str.2005.11.014

[b11] Zhao G. . Mature HIV-1 capsid structure by cryo-electron microscopy and all-atom molecular dynamics. Nature 497, 643–646 (2013). 23719463 10.1038/nature12162 PMC3729984

[b12] Mulholland A. J. Modelling enzyme reaction mechanisms, specificity and catalysis. Drug Discov. Today 10, 1393–1402 (2005). 16253878 10.1016/S1359-6446(05)03611-1

[b13] Rudack T., Xia F., Schlitter J., Kötting C. & Gerwert K. Ras and GTPase-activating protein (GAP) drive GTP into a precatalytic state as revealed by combining FTIR and biomolecular simulations. Proceedings of the National Academy of Sciences 109, 15295–15300 (2012). 10.1073/pnas.1204333109 PMC3458370 22949691

[b14] Bernardi R. C. & Pascutti P. G. Hybrid QM/MM molecular dynamics study of benzocaine in a membrane environment: how does a quantum mechanical treatment of both anesthetic and lipids affect their interaction. J. Chem. Theor. Comp. 8, 2197–2203 (2012). 10.1021/ct300213u 26588952

[b15] Bucher D. . Polarization effects and charge transfer in the KcsA potassium channel. Biophys. Chem. 124, 292–301 (2006). 16737771 10.1016/j.bpc.2006.04.008

[b16] Goh B. C. . Computational methodologies for real-space structural refinement of large macromolecular complexes. *Annu. Rev. Biophys*. 45, 253–278 (2016). 10.1146/annurev-biophys-062215-011113 PMC5526348 27145875

[b17] Bernardi R. C., Melo M. C. R. & Schulten K. Enhanced sampling techniques in molecular dynamics simulations of biological systems. Biochim. Biophys. Acta 1850, 872–877 (2015). 25450171 10.1016/j.bbagen.2014.10.019 PMC4339458

[b18] Jo S., Kim T., Iyer V. G. & Im W. CHARMM-GUI: A web-based graphical user interface for CHARMM. J. Comp. Chem. 29, 1859–1865 (2008). 18351591 10.1002/jcc.20945

[b19] Phillips J. C. . Scalable molecular dynamics with NAMD. J. Comp. Chem. 26, 1781–1802 (2005). 16222654 10.1002/jcc.20289 PMC2486339

[b20] Humphrey W., Dalke A. & Schulten K. VMD - Visual Molecular Dynamics. J. Mol. Graphics 14, 33–38 (1996). 10.1016/0263-7855(96)00018-5 8744570

[b21] Berman H. M. . The protein data bank. Nucleic Acids Res. 28, 235–242 (2000). 10592235 10.1093/nar/28.1.235 PMC102472

[b22] Sali A. & Blundell T. L. Comparative protein modelling by satisfaction of spatial restraints. J. Mol. Biol. 234, 779 (1993). 8254673 10.1006/jmbi.1993.1626

[b23] Leaver-Fay A. . Rosetta3: an object-oriented software suite for the simulation and design of macromolecules. Meth. Enzym. 487, 545 (2011). 21187238 10.1016/B978-0-12-381270-4.00019-6 PMC4083816

[b24] Zhang J. . MUFOLD: A new solution for protein 3D structure prediction. Proteins: Struct., Func., Bioinf. 78, 1137–1152 (2010). 10.1002/prot.22634 PMC2885889 19927325

[b25] Arnold K., Bordoli L., Kopp J. & Schwede T. The swiss-model workspace: a web-based environment for protein structure homology modelling. Bioinformatics 22, 195–201 (2006). 16301204 10.1093/bioinformatics/bti770

[b26] Roy A., Kucukural A. & Zhang Y. I-tasser: a unified platform for automated protein structure and function prediction. Nat. Protocols 5, 725–738 (2010). 20360767 10.1038/nprot.2010.5 PMC2849174

[b27] Mayne C. G., Saam J., Schulten K., Tajkhorshid E. & Gumbart J. C. Rapid parameterization of small molecules using the Force Field Toolkit. J. Comp. Chem. 34, 2757–2770 (2013). 24000174 10.1002/jcc.23422 PMC3874408

[b28] Olsson M. H., Søndergaard C. R., Rostkowski M. & Jensen J. H. Propka3: Consistent treatment of internal and surface residues in empirical pKa predictions. J. Chem. Theor. Comp. 7, 525–537 (2011). 10.1021/ct100578z 26596171

[b29] Berendsen H. J. C., Postma J. P. M., van Gunsteren W. F., DiNola A. & Haak J. R. Molecular dynamics with coupling to an external bath. J. Chem. Phys. 81, 3684–3690 (1984).

[b30] Izrailev S., Stepaniants S., Balsera M., Oono Y. & Schulten K. Molecular dynamics study of unbinding of the avidin-biotin complex. Biophys. J. 72, 1568–1581 (1997). 9083662 10.1016/S0006-3495(97)78804-0 PMC1184352

[b31] Trabuco L. G., Villa E., Mitra K., Frank J. & Schulten K. Flexible fitting of atomic structures into electron microscopy maps using molecular dynamics. Structure 16, 673–683 (2008). 18462672 10.1016/j.str.2008.03.005 PMC2430731

[b32] Trabuco L. G., Villa E., Schreiner E., Harrison C. B. & Schulten K. Molecular Dynamics Flexible Fitting: A practical guide to combine cryo-electron microscopy and X-ray crystallography. Methods 49, 174–180 (2009). 19398010 10.1016/j.ymeth.2009.04.005 PMC2753685

[b33] Stone J. E., Gullingsrud J., Grayson P. & Schulten K. A system for interactive molecular dynamics simulation. In Hughes J. F. & Séquin C. H. (eds.) 2001 ACM Symposium on Interactive 3D Graphics 191–194 (ACM SIGGRAPH, New York, 2001).

[b34] Onuchic J. N., Luthey-Schulten Z. & Wolynes P. G. Theory of protein folding: The energy landscape perspective. Annu. Rev. Phys. Chem. 48, 545–600 (1997). 9348663 10.1146/annurev.physchem.48.1.545

[b35] Ma W. & Schulten K. Mechanism of substrate translocation by a ring-shaped ATPase motor at millisecond resolution. J. Am. Chem. Soc. 137, 3031–3040 (2015). 25646698 10.1021/ja512605w PMC4393844

[b36] Jayachandran G., Vishal V. & Pande V. S. Using massively parallel simulation and markovian models to study protein folding: examining the dynamics of the villin headpiece. J. Chem. Phys. 124, 164902 (2006). 16674165 10.1063/1.2186317

[b37] Michaud-Agrawal N., Denning E. J., Woolf T. B. & Beckstein O. MDAnalysis: A toolkit for the analysis of molecular dynamics simulations. J. Comp. Chem. 32, 2319–2327 (2011). 21500218 10.1002/jcc.21787 PMC3144279

[b38] Levine B. G., Stone J. E. & Kohlmeyer A. Fast analysis of molecular dynamics trajectories with graphics processing units-radial distribution function histogramming. J. Comp. Phys. 230, 3556–3569 (2011). 10.1016/j.jcp.2011.01.048 PMC3085256 21547007

[b39] Roe D. R. & Cheatham T. E. III PTRAJ and CPPTRAJ: Software for Processing and Analysis of Molecular Dynamics Trajectory Data. J. Chem. Theor. Comp. 9, 3084–3095 (2013). 10.1021/ct400341p 26583988

[b40] Stone J. E., McGreevy R., Isralewitz B. & Schulten K. GPU-accelerated analysis and visualization of large structures solved by molecular dynamics flexible fitting. Faraday Discuss. 169, 265–283 (2014). 25340325 10.1039/c4fd00005f PMC4208074

[b41] Perryman A. L., Lin J.-H. & McCammon J. A. HIV-1 protease molecular dynamics of a wild-type and of the V82F/I84V mutant: Possible contributions to drug resistance and a potential new target site for drugs. Prot. Sci. 13, 1108–1123 (2004). 10.1110/ps.03468904 PMC2280056 15044738

[b42] Hornak V., Okur A., Rizzo R. C. & Simmerling C. HIV-1 protease flaps spontaneously open and reclose in molecular dynamics simulations. Proc. Natl. Acad. Sci. USA 103, 915–920 (2006). 16418268 10.1073/pnas.0508452103 PMC1347991

[b43] Costa M. G. . Impact of M36I polymorphism on the interaction of HIV-1 protease with its substrates: insights from molecular dynamics. BMC Genomics 15, S5 (2014). 25573486 10.1186/1471-2164-15-S7-S5 PMC4243740

[b44] Cann I., Bernardi R. C. & Mackie R. I. Cellulose degradation in the human gut: Ruminococcus champanellensis expands the cellulosome paradigm. Environ Microbiol 18, 307–310 (2016). 26781441 10.1111/1462-2920.13152

[b45] Beckham G. T., Bomble Y. J., Bayer E. A., Himmel M. E. & Crowley M. F. Applications of computational science for understanding enzymatic deconstruction of cellulose. Curr. Opin. Biotech. 22, 231–238 (2011). 21168322 10.1016/j.copbio.2010.11.005

[b46] Bernardi R. C., Cann I. & Schulten K. Molecular dynamics study of enhanced Man5B enzymatic activity. Biotechnol. Biofuels 7, 1–8 (2014). 24976862 10.1186/1754-6834-7-83 PMC4074406

[b47] Janosi L. & Ceccarelli M. The gating mechanism of the human aquaporin 5 revealed by molecular dynamics simulations. PloS ONE 8, e59897 (2013). 23565173 10.1371/journal.pone.0059897 PMC3614956

[b48] Stone J. E. . Atomic detail visualization of photosynthetic membranes with GPU-accelerated ray tracing. *Parallel Computing* (2016). In Press. 10.1016/j.parco.2015.10.015 PMC4890717 27274603

[b49] Schoeler C. . Ultrastable cellulosome-adhesion complex tightens under load. Nat. Commun. 5, 5635 (2014). 25482395 10.1038/ncomms6635 PMC4266597

[b50] Schoeler C. . Mapping mechanical force propagation through biomolecular complexes. Nano Lett. 15, 7370–7376 (2015). 26259544 10.1021/acs.nanolett.5b02727 PMC4721519

[b51] Stone J. E., Vandivort K. L. & Schulten K. GPU-accelerated molecular visualization on petascale supercomputing platforms. In *Proceedings of the 8th International Workshop on Ultrascale Visualization,* UltraVis ’13, 6:1–6:8 (ACM, New York, NY, USA, 2013).

[b52] Tan T. . Video quality evaluation methodology and verification testing of HEVC compression performance. Circuits and Systems for Video Technology, IEEE Transactions on 26, 76–90 (2016).

[b53] Brooks B. R. . CHARMM: A program for macromolecular energy, minimization, and dynamics calculations. J. Comp. Chem. 4, 187–217 (1983).

[b54] Best R. B. . Optimization of the additive charmm all-atom protein force field targeting improved sampling of the backbone *ϕ, ψ* and side-chain *χ*_1_ and *χ*_2_ dihedral angles. J. Chem. Theor. Comp. 8, 3257–3273 (2012). 10.1021/ct300400x PMC3549273 23341755

[b55] MacKerell A. D. Jr. . All-atom empirical potential for molecular modeling and dynamics studies of proteins. J. Phys. Chem. B 102, 3586–3616 (1998). 24889800 10.1021/jp973084f

[b56] Jorgensen W. L., Chandrasekhar J., Madura J. D., Impey R. W. & Klein M. L. Comparison of simple potential functions for simulating liquid water. J. Chem. Phys. 79, 926–935 (1983).

[b57] Darden T., York D. & Pedersen L. G. Particle mesh Ewald: An *N* · log(*N*) method for Ewald sums in large systems. J. Chem. Phys. 98, 10089–10092 (1993).

[b58] Isralewitz B., Gao M. & Schulten K. Steered molecular dynamics and mechanical functions of proteins. Curr. Opin. Struct. Biol. 11, 224–230 (2001). 11297932 10.1016/s0959-440x(00)00194-9

[b59] Ieong P. U. . Progress towards automated Kepler scientific workflows for computer-aided drug discovery and molecular simulations. Proc. Comp. Sci. 29, 1745–1755 (2014). 10.1016/j.procs.2014.05.159 PMC5796787 29399238

[b60] Tanner D.E., Chan K.Y., Phillips J.C., & Schulten K. Parallel generalized Born implicit solvent calculations with NAMD. J. Chem. Theo. Comp. 7, 3635–3642 (2011). 10.1021/ct200563j PMC3222955 22121340

