# Supplementary material for: QwikMD — Integrative Molecular Dynamics Toolkit for Novices and Experts
Source: Sci Rep. 2016 May 24;6:26536. doi: 10.1038/srep26536 (PMC4877583; doi:10.1038/srep26536)

QwikMD – Integrative Molecular Dynamics Toolkit for Novices and Experts

João V. Ribeiro[1,2*], Rafael C. Bernardi[1,2*], Till Rudack[1*], John E. Stone[1], James C. Phillips[1], Peter L. Freddolino[3], Klaus Schulten[1,2,4**]

*These authors contributed equally to this work and are presented in alphabetical order.
1. Beckman Institute for Advanced Science and Technology, University of Illinois at Urbana-Champaign, USA
2. Energy Biosciences Institute, University of Illinois at Urbana-Champaign, USA
3. Department of Biological Chemistry, University of Michigan Medical School, Ann Arbor, USA
4. Department of Physics, University of Illinois at Urbana-Champaign, USA
** Corresponding author e-mail: kschulte@ks.uiuc.edu

Supplementary Note S1

Using Google Scholar data, plots presented at Figure 1B are based on the number of citations per year of the following manuscripts for each software.

AMBER:

• Case, D. A., Cheatham, T. E., Darden, T., Gohlke, H., Luo, R., Merz, K. M., ... & Woods, R. J. (2005). *The Amber biomolecular simulation programs*. Journal of Computational Chemistry, 26(16), 1668-1688.

• Pearlman, D. A., Case, D. A., Caldwell, J. W., Ross, W. S., Cheatham, T. E., DeBolt, S., ... & Kollman, P. (1995). *AMBER, a package of computer programs for applying molecular mechanics, normal mode analysis, molecular dynamics and free energy calculations to simulate the structural and energetic properties of molecules.* Computer Physics Communications, 91(1), 1-41.

• Weiner, P. K., & Kollman, P. A. (1981). *AMBER: Assisted model building with energy refinement. A general program for modeling molecules and their interactions.* Journal of Computational Chemistry, 2(3), 287-303.

CHARMM:

• Brooks, B. R., Brooks, C. L., MacKerell, A. D., Nilsson, L., Petrella, R. J., Roux, B., ... & Caflisch, A. (2009). *CHARMM: the biomolecular simulation program.* Journal of Computational Chemistry, 30(10), 1545-1614.

• Brooks, B. R., Bruccoleri, R. E., Olafson, B. D., States, D. J., Swaminathan, S., & Karplus, M. (1983). CHARMM: *A program for macromolecular energy, minimization, and dynamics calculations.* Journal of Computational Chemistry,4(2), 187-217.

GROMACS:

• Pronk, S., Páll, S., Schulz, R., Larsson, P., Bjelkmar, P., Apostolov, R., ... & Hess, B. (2013). *GROMACS 4.5: a high-throughput and highly parallel open source molecular simulation toolkit.* Bioinformatics, 29(7), 845-854.

• Hess, B., Kutzner, C., Van Der Spoel, D., & Lindahl, E. (2008). *GROMACS 4: algorithms for highly efficient, load-balanced, and scalable molecular simulation.* Journal of Chemical Theory and Computation, 4(3), 435-447.

• Van Der Spoel, D., Lindahl, E., Hess, B., Groenhof, G., Mark, A. E., & Berendsen, H. J. (2005). *GROMACS: fast, flexible, and free.* Journal of Computational Chemistry, 26(16), 1701-1718.

• Lindahl, E., Hess, B., & Van Der Spoel, D. (2001). *GROMACS 3.0: a package for molecular simulation and trajectory analysis.* Molecular Modeling Annual, 7(8), 306-317.

• Berendsen, H. J., van der Spoel, D., & van Drunen, R. (1995). *GROMACS: a message passing parallel molecular dynamics implementation*. Computer Physics Communications, 91(1), 43-56.

LAMMPS:

• Plimpton, S. (1995). *Fast parallel algorithms for short-range molecular dynamics.* Journal of Computational Physics, 117(1), 1-19.

NAMD:

• Phillips, J. C., Braun, R., Wang, W., Gumbart, J., Tajkhorshid, E., Villa, E., ... & Schulten, K. (2005). *Scalable molecular dynamics with NAMD.* Journal of Computational Chemistry, 26(16), 1781-1802.

• Kalé, L., Skeel, R., Bhandarkar, M., Brunner, R., Gursoy, A., Krawetz, N., ... & Schulten, K. (1999). *NAMD2: greater scalability for parallel molecular dynamics.* Journal of Computational Physics, 151(1), 283-312.

VMD:

• Humphrey, W., Dalke, A., & Schulten, K. (1996). *VMD: visual molecular dynamics.* Journal of Molecular Graphics, 14(1), 33-38.

Supplementary Figure S2

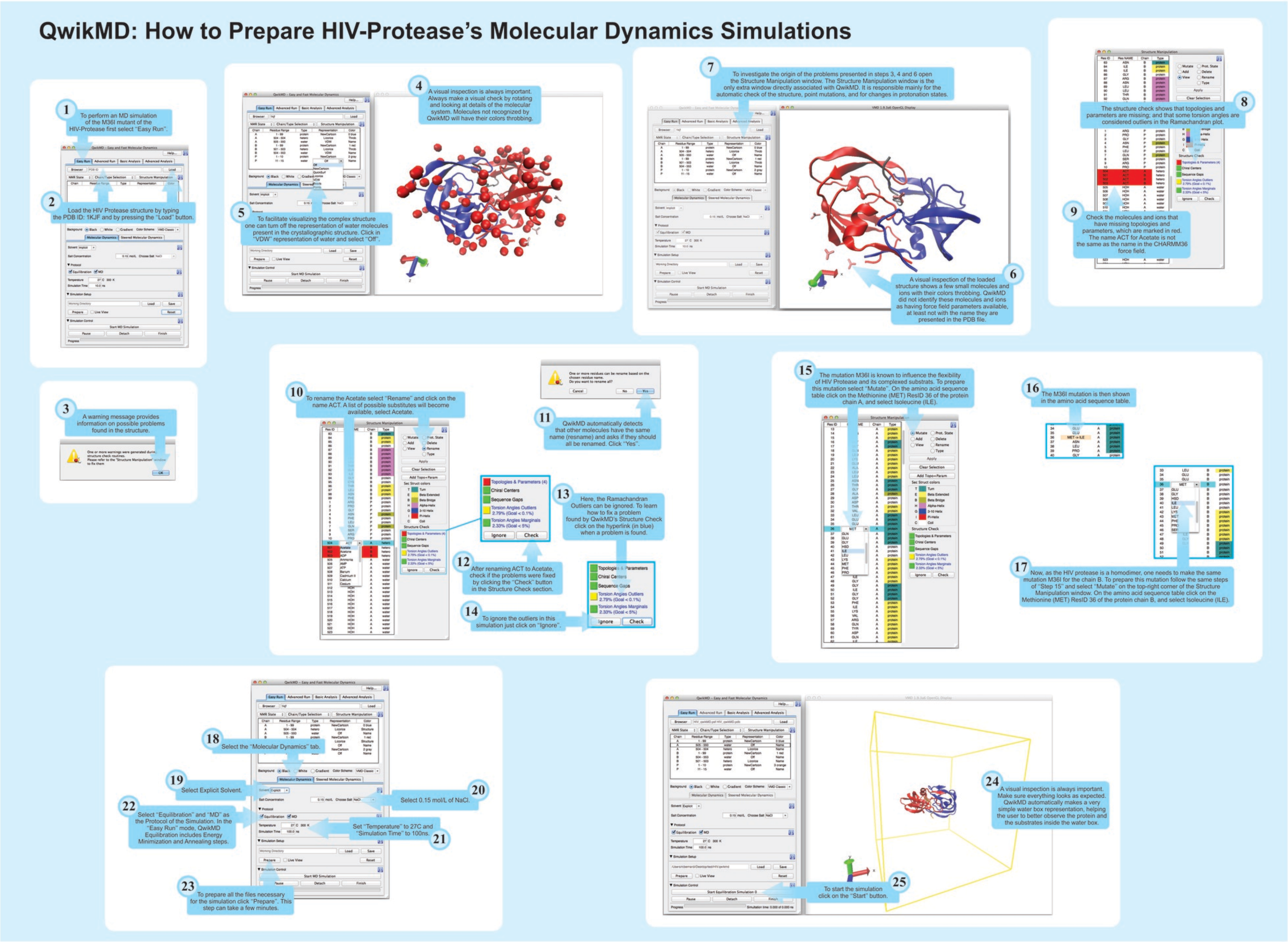

Supplementary Figure S3

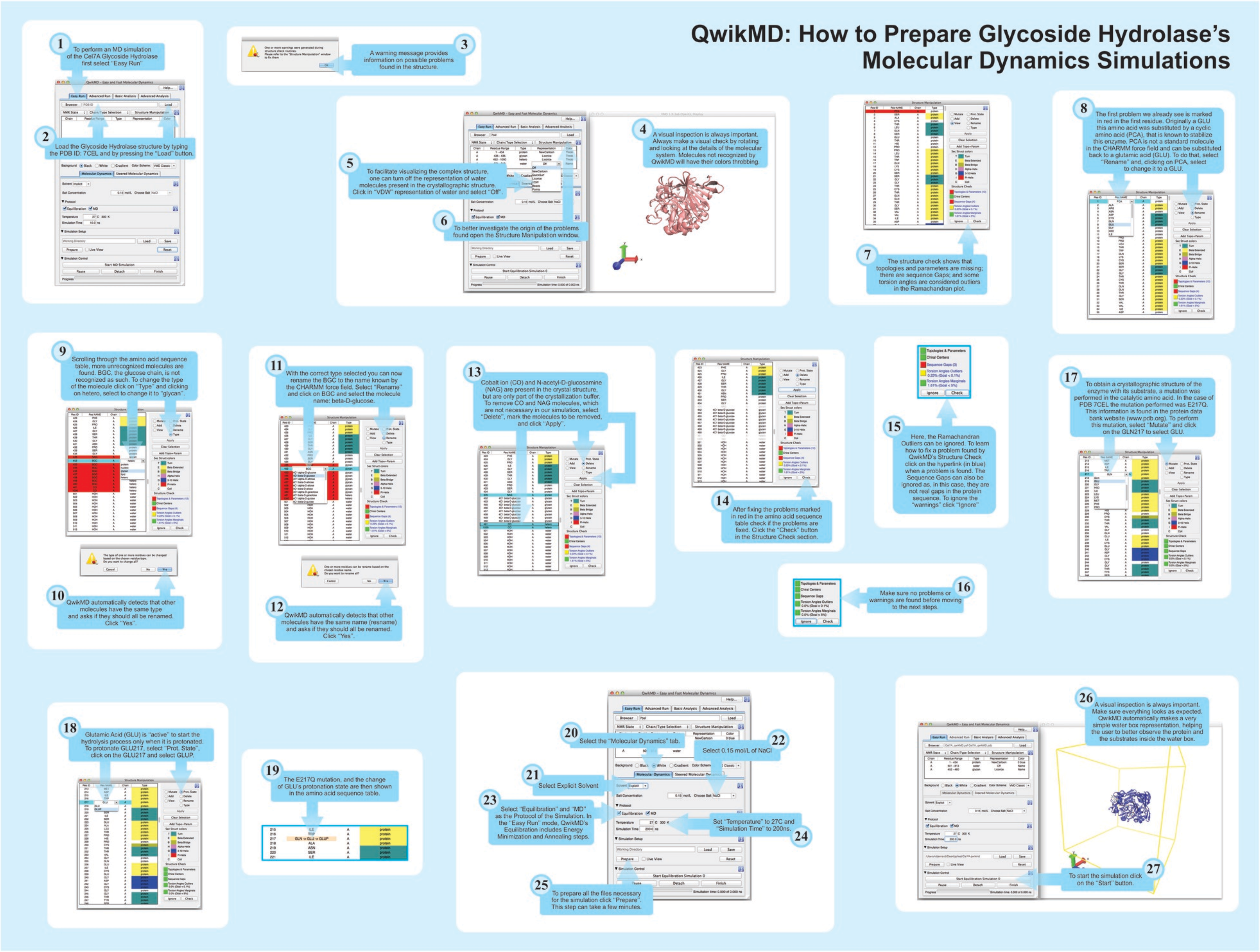

Supplementary Figure S4

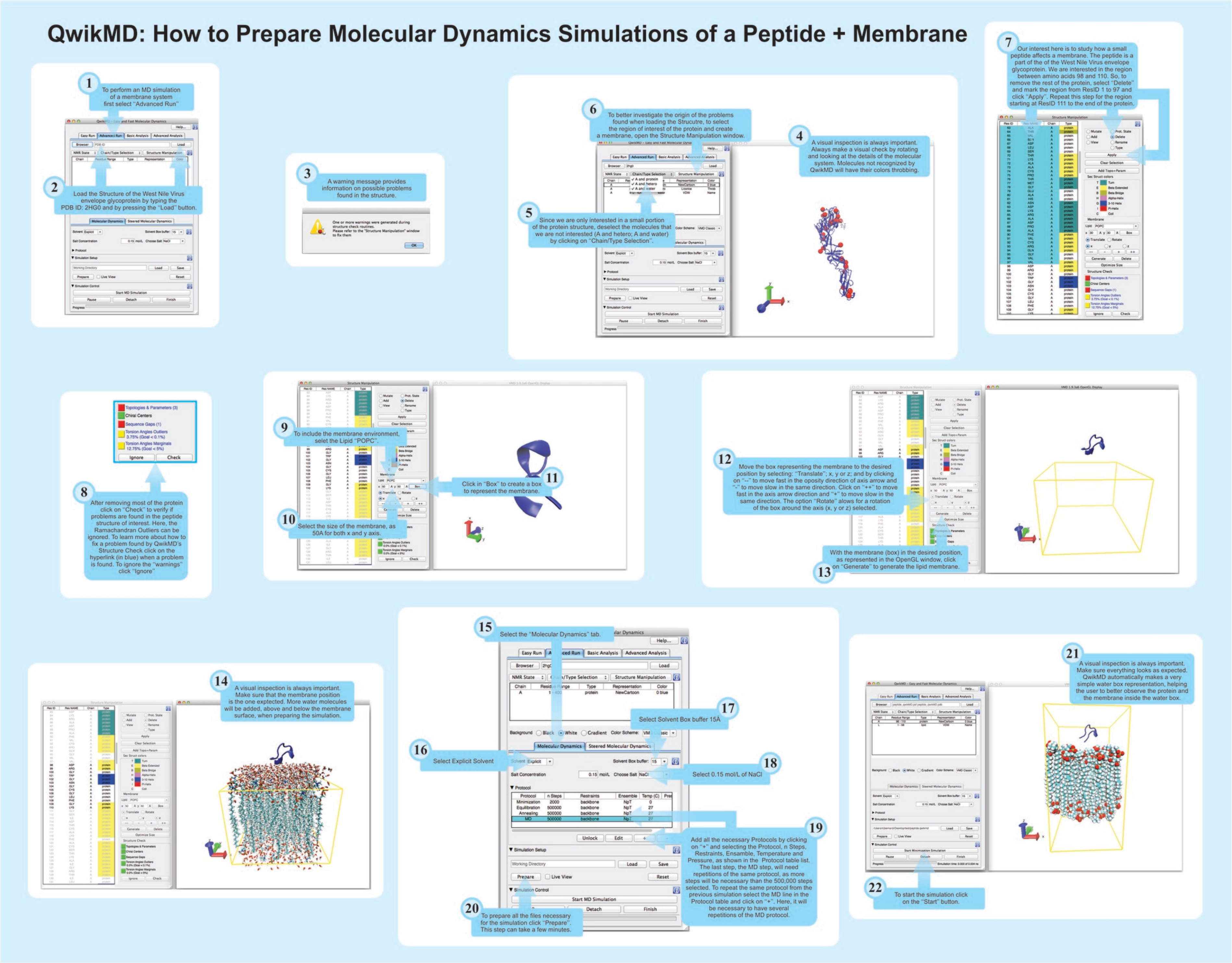

Supplementary Figure S5

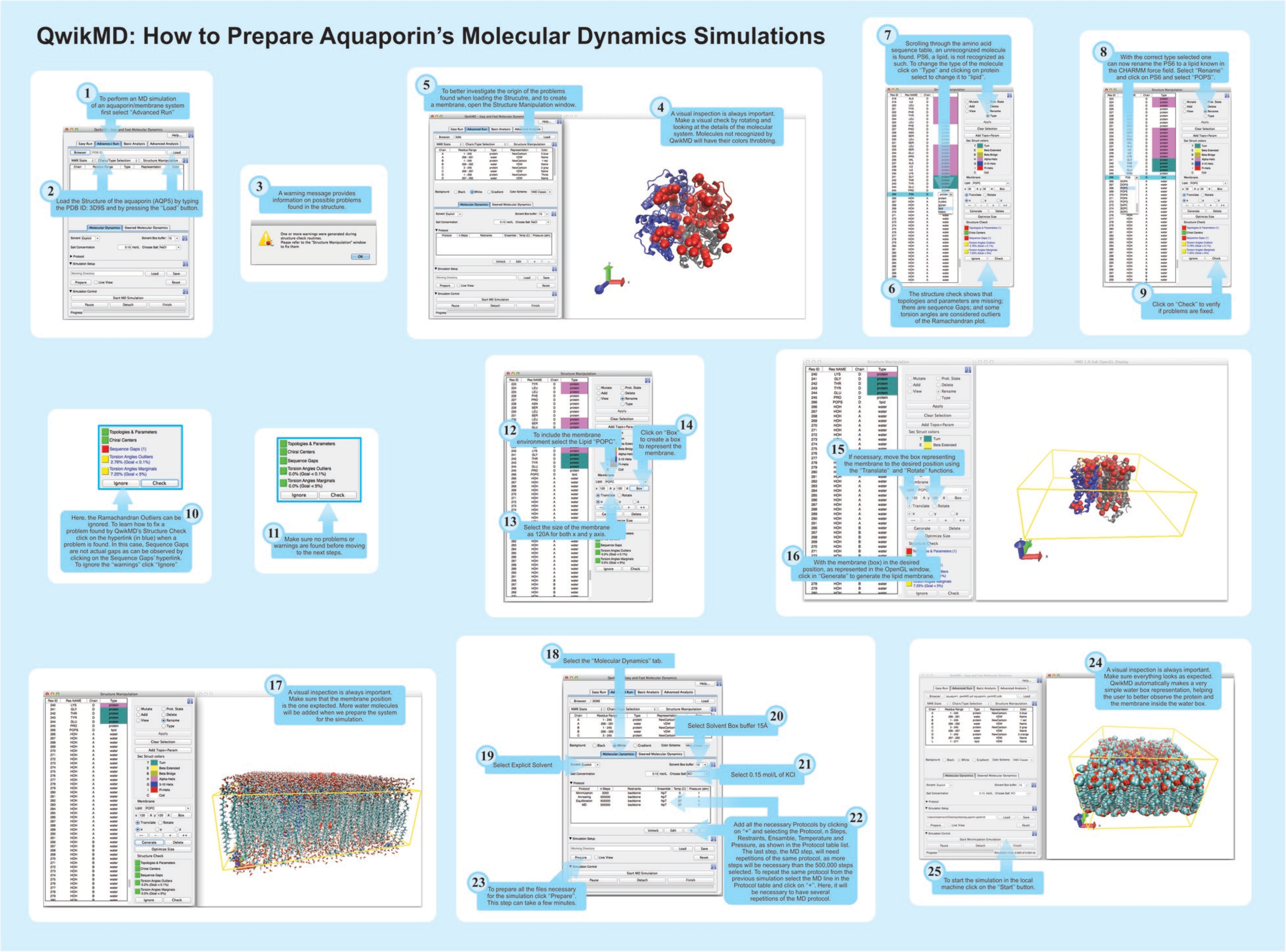

Supplementary Figure S6

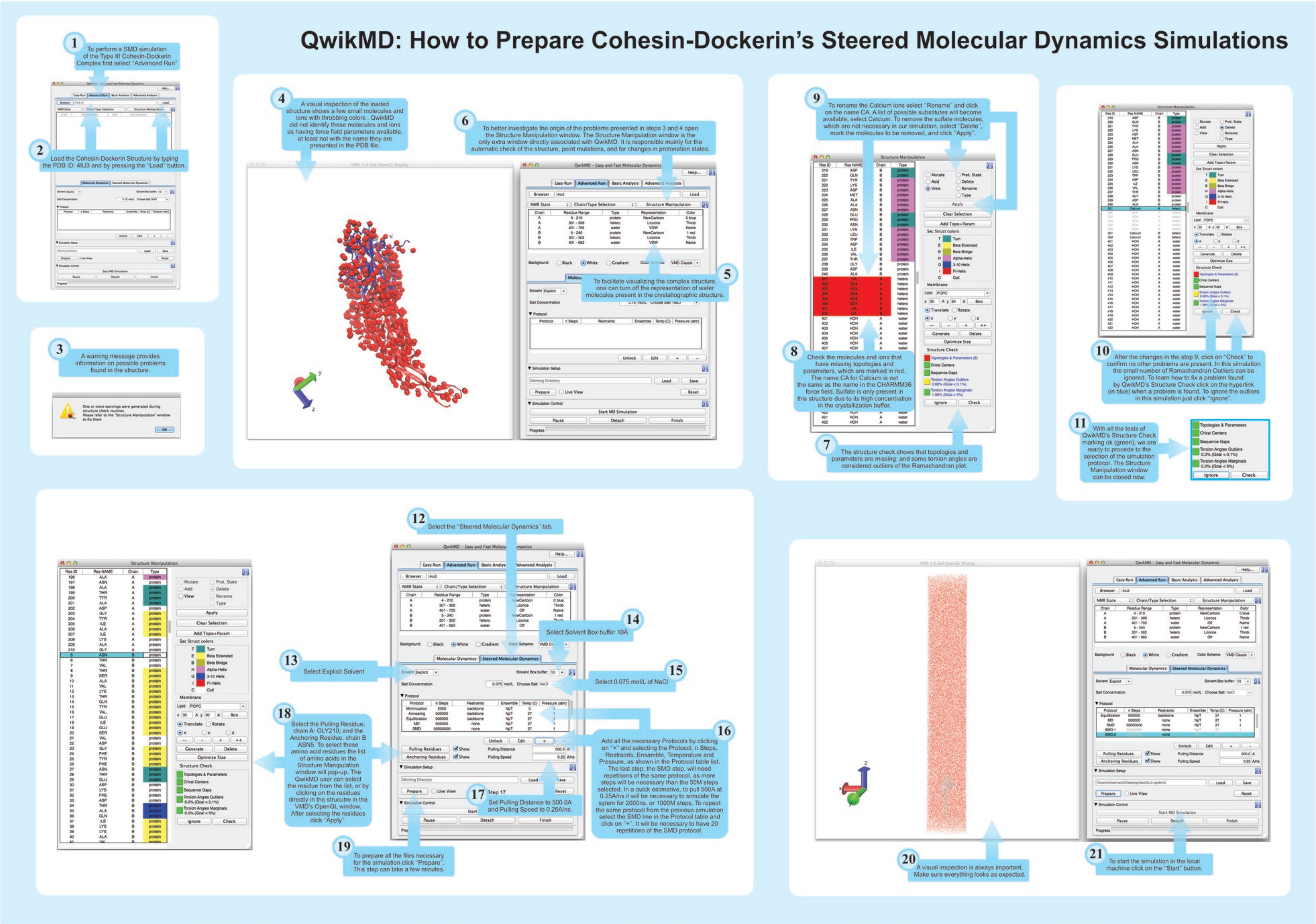

Supplement: Supplementary Information [file srep26536-s1.pdf]
